# A National Survey of Community Pharmacists’ Viewpoints About Pharmacovigilance and Adverse Drug Reaction Reporting in Saudi Arabia

**DOI:** 10.3389/fphar.2022.819551

**Published:** 2022-05-26

**Authors:** Mona Y. Alsheikh, Moudi M. Alasmari

**Affiliations:** ^1^ Department of Clinical Pharmacy, College of Pharmacy, Taif University, Taif, Saudi Arabia; ^2^ College of Medicine, King Saud bin Abdulaziz University for Health Sciences (KSAU-HS), Jeddah, Saudi Arabia; ^3^ King Abdullah International Medical Research Center (KAIMRC), Jeddah, Saudi Arabia

**Keywords:** adverse drug reactions, community pharmacists, healthcare, pharmacovigilance, Saudi Arabia

## Abstract

This study assessed the knowledge, attitudes, and practices of community pharmacists regarding pharmacovigilance and adverse drug reaction (ADR) reporting system in Saudi Arabia. A cross-sectional survey of community pharmacists from different regions in Saudi Arabia was performed through convenience sampling between November 2020 and January 2021. The responses were received from 1,172 community pharmacists. Most respondents (86.7%) were familiar with the National Pharmacovigilance and Drug Safety Center, and 830 (70.8%) knew about the ADR reporting form. The majority (94%) of the respondents agreed with the importance of reporting ADRs for patient care and national health. Although 92.2% of the participants asked their patients about ADRs, 90.2% agreed that more training programs are required to be organized by the Saudi Food and Drug Authority for healthcare professionals on the ADR detection and reporting system. Analgesic agents were the most common drug category for which ADRs were reported (67.4%). The majority (92.1%) of ADRs reportedly occurred in patients with chronic diseases. The study concluded that most community pharmacists in Saudi Arabia are knowledgeable and have good attitudes and practices regarding pharmacovigilance and ADR reporting.

## Introduction

Medication safety is an important global concern, and it is monitored and assessed using pharmacovigilance systems. Pharmacovigilance is defined as the activities linked to the detection, assessment, understanding, and prevention of adverse drug reactions (ADRs) (World Health Organization, 2002). The Saudi Food and Drug Authority (SFDA) has established the Pharmacovigilance System for monitoring drug safety in Saudi Arabia under the guidance of the National Pharmacovigilance and Drug Safety Center (Alharf et al., 2018). The pharmacovigilance activities include the evaluation of ADRs, detection of signals, assessment of risks, evaluation of vaccine safety, and provision of periodic safety update reports (Alharf et al., 2018; Alwhaibi et al., 2020).

According to the World Health Organization (WHO), an ADR can be defined as any unpredictable, unintended effect of medication that is directly harmful at regular doses (Coleman and Pontefract, 2016). Globally, ADRs are recognized as the foremost cause of morbidity and mortality (Wu et al., 2010; Silva et al., 2021). They adversely affect patients and their quality of life, leading to severe consequences such as hospitalizations, disabilities, life-threatening conditions, or even death. They also increase healthcare costs and have a negative impact on the healthcare systems (Bénard-Laribière et al., 2015; Geer et al., 2016; Veeren and Weiss, 2017; Patton and Borshoff, 2018; Alayed et al., 2019).

Giardina et al. (2018) reported an increase in hospitalization rates due to ADRs in Italy. In England, there was a 53.4% increase in the emergency admissions due to ADRs in 2014/2015 when compared to their frequency in 2008/2009 (Veeren and Weiss, 2017). In France, the incidence rate of patient hospitalizations because of ADRs was 3.6% (Bénard-Laribière et al., 2015). The incidence of ADR-related hospitalizations in Saudi Arabia has been described previously (Aljadhey et al., 2013; Alayed et al., 2019). Aljadhey et al. (2016) reported an ADR incidence of 6.1 per 100 admissions in Saudi Arabia. The medical burden of severe and fatal ADRs is high. In the United States of America and Sweden, fatal ADRs are the sixth and seventh leading causes of mortality, respectively (Lazarou et al., 1998; Wester et al., 2008). In Finland, 5% of deaths in a university central hospital was reported to be drug related (Juntti-Patinen and Neuvonen, 2002). Early detection and prevention of ADRs are urgent and should be a common goal for healthcare providers. Although there are limited methods for monitoring ADRs, they have a significant clinical impact.

Pharmacovigilance plays an important role in ensuring the safety of medications through the detection, assessment, and understanding of the adverse impact of pharmaceutical products (Härmark and Van Grootheest, 2008; Kumar et al., 2011). The most influential pharmacovigilance activity is the spontaneous reporting by healthcare practitioners (such as physicians, pharmacists, and nurses) of suspected ADRs that had not been identified during premarketing clinical trials (Güner and Ekmekci, 2019). National systems for reporting drug adverse reactions exist in almost every country. The FDA Adverse Event Reporting System (FAERS) was launched in 1998 in the United States. Healthcare professionals use the FAERS database to study the safety-related drug issues. Reporting ADRs through pharmacovigilance has been increasingly gaining attention globally (Sonawane and Hansen, 2015).

Underreporting is a significant challenge in pharmacovigilance programs (Alharf et al., 2018). The contribution of Saudi Arabia, along with other Middle Eastern countries, to global safety reporting is only 0.6% (Ahmad, 2014). This confirms that underreporting in Saudi Arabia is a significant concern, which may be attributed to the lack of knowledge and training in healthcare providers about pharmacovigilance and medication safety maintenance (Ahmad, 2014; Alshammari et al., 2017; AlShammari and Almoslem, 2018).

Studies involving community pharmacists in Saudi Arabia and assessing their understanding of ADR reporting and pharmacovigilance awareness are limited (Mahmoud et al., 2014; Ali et al., 2018; Cheema et al., 2019). The lack of pharmacovigilance as a subject of study in healthcare institutions is one of the primary reasons for healthcare providers’ lack of knowledge of pharmacovigilance and ADR reporting (Mahmoud et al., 2014; Almandil, 2016). A systematic review has indicated that students’ qualifications were inadequate in terms of describing ADRs or performing pharmacovigilance (Reumerman et al., 2018). However, several previous studies have indicated that pharmacy students had a higher level of knowledge than students from other healthcare schools (Sivadasan et al., 2014; Khan et al., 2015).

A community pharmacist remains the most easily accessible healthcare professional to the public and is likely the first person approached for drug information (Daly et al., 2020). The present study assessed community pharmacists’ knowledge, attitudes, and practices regarding pharmacovigilance and ADR reporting in Saudi Arabia. This study confirms the presence of a certain educational gap in community pharmacists and argues for the need to facilitate specific educational programs to promote safe practices and support the pharmacovigilance environment for future community pharmacists.

## Materials and Methods

### Study Design and Participants

A survey-based cross-sectional study was conducted from November 2020 to January 2021 in a convenience sample of community pharmacists from different regions of Saudi Arabia to assess their knowledge, attitudes, and practices regarding pharmacovigilance and ADR reporting.

### Inclusion and Exclusion Criteria

All the registered community pharmacists in Saudi Arabia, regardless of sex and nationality, were included in this study. Community pharmacists were recruited using convenience sampling. This study excluded pharmacy technicians, hospital pharmacists, and those community pharmacists who were not registered by the Saudi Commission for Health Specialties (SCFHS).

### Study Procedure

A self-administered Internet-based survey was conducted. A questionnaire was created to meet the specific objectives of this study, which was divided into four sections: demographic characteristics, knowledge, attitudes, and practices. The participants were asked to score statements based on how well they described their knowledge and training regarding the ways of ADR reporting to the SFDA using a 5-point Likert scale, starting from “not at all” = 1, “not well” = 2, “average” = 3, “well” = 4, and “very well” = 5. The score ranged 4–5, 2–3, 1–2, and <1, indicating good, fair, unsatisfactory, and poor knowledge, respectively. The questions were derived from the relevant literature and reviewed by two expert academic pharmacists from the College of Pharmacy of the Taif University. The modifications were made based on the reviewers’ suggestions. The questionnaire was checked for face and content validity by five pharmacy staff members of the Department of Clinical Pharmacy at the Taif University College of Pharmacy. A pilot study was conducted with 15 community pharmacists experienced in pharmacy practice and research backgrounds. The questionnaire was written in both English and Arabic to avoid misunderstanding. The survey was designed to be simple, such that the participants could complete it in the shortest possible time, approximately 10–15 min.

### Data Collection

A self-administered Internet-based survey was used to collect the data from pharmacists in Saudi Arabia from November 2020 to January 2021. The Google Forms survey was designed for online completion. The questionnaire link was sent to key persons in the pharmacy groups and *via* WhatsApp messenger to the Saudi Arabian community pharmacists’ professional groups.

### Ethical Considerations

The study received ethical approval from the Research and Ethics Committees at the Taif University (reference number: 42–144, and King Abdullah International Medical Research Center (IRB number: NRJ21J/195/08). All the participants provided informed consent, and confidentiality and anonymity were ensured. The data were anonymously downloaded in an Excel document from the Google Forms. No information was requested that could identify the participants.

### Statistical Analyses

Microsoft Excel 2016 and IBM^®^ SPSS Statistics version 28.0 (IBM Corp., Armonk, NY, United States) were used to perform the statistical analyses. Descriptive statistics were used and presented as number (N) and percentage (%) to describe the knowledge, attitudes, and practices of the respondents related to pharmacovigilance and ADR reporting. The Chi-squared and Fisher’s exact tests were performed to assess the differences in proportions, where appropriate. The statistical significance level was set a priori at *p < 0.05*.

## Results

### Demographic Characteristics

The questionnaire was completed by 1,172 of the 1,231 contacted community pharmacists, indicating a response rate of 95.2%. The demographic profiles of the respondents are provided in Table 1. The majority of the respondents were 24–35 years old (970, 82.8%), men (1,126, 96.1%), non-Saudi (1,078, 92.0%), bachelor degree holders (956, 81.6%), professionally classified as pharmacists (665, 56.7%), and working in the Western region of Saudi Arabia (566, 48.3%). Most worked in cities (1,078, 92.0%) and chain pharmacies (1,127, 96.2%). With respect to the employment status and experience, most had a full-time contract (1,107, 94.5%) and more than half (611, 52.1%) had 5–10 years of experience and worked in an evening shift (604, 51.5%).

**TABLE 1:** Demographic profile of the sample of community pharmacists.

Items	Measures	Frequency (n = 1,172)	Percentage (%)
Age	24–35 years	970	82.8
	36–45 years	177	15.1
	46–55 years	21	1.8
	>55 years	4	0.3
Gender	Male	1,126	96.1
	Female	46	3.9
Nationality	Saudi	94	8.0
	Non-Saudi	1,078	92.0
Educational level	BPharm	956	81.6
	PharmD	116	9.9
	Master	18	1.5
	PhD	82	7.0
Professional classification	Pharmacist	665	56.7
	Senior pharmacist	444	37.9
	Consultant pharmacist	63	5.4
Region	Western region	566	48.3
	Central Region	280	23.9
	Southern Region	214	18.3
	Eastern Region	83	7.1
	Northern Region	29	2.5
Pharmacy location	City	1,078	92.0
	Village	94	8.0
Type of community pharmacy	Chain pharmacy	1,127	96.2
	Independent pharmacy	45	3.8
Employment contract status	Full time	1,107	94.5
	Part time	62	5.3
	Temporary/casual	3	0.3
Years of experience	<5 years	312	26.6
	5–10 years	611	52.1
	11–20 years	238	20.3
	>20 years	11	0.9
Usual shift	Evening (8 a.m.–4 p.m.)	604	51.5
	Morning (4 p.m.–2 a.m.)	319	27.2
	Night (12 a.m.–8 a.m.)	249	21.2

### Knowledge of Community Pharmacists Regarding Pharmacovigilance and Adverse Drug Reaction Reporting

The knowledge of the respondents regarding pharmacovigilance and ADR reporting is shown in Table 2. Half of the participants had indicated their understanding of the term “pharmacovigilance” on a 4-point Likert scale as very familiar (596, 50.9%) and the term “adverse effects” as also very familiar (649, 55.4%). More than half had indicated that not all serious adverse effects are not known before the drugs become marketed (663, 56.6%). The majority knew about the National Pharmacovigilance and Drug Safety Center administered by the SFDA (1,016, 86.7%). They were familiar with the ADR reporting form for healthcare professionals (Form No. ADR-1) (830, 70.8%), knew where to obtain it (744, 63.5%), and were aware of how the reporting form should be submitted (951, 81.1%). Less than half rated their knowledge and training about reporting ADRs to the SFDA at 4 of 5 points (463, 39.5%).

**TABLE 2:** Pharmacovigilance and ADR reporting related knowledge of the respondents.

Variable		Frequency (*n* = 1,172)	Percentage (%)
How familiar are you with the term “pharmacovigilance”?	Very familiar—I have a complete understanding	596	50.9
	Familiar—I have a basic understanding	489	41.7
	Heard of the term—cannot define it	56	4.8
	Never heard of the term	31	2.6
How familiar are you with the term “adverse effect”?	Very familiar—I have a complete understanding	649	55.4
	Familiar—I have a basic understanding	446	38.1
	Heard of the term—cannot define it	53	4.5
	Never heard of the term	24	2.0
Do you think all serious adverse effects are known before a drug is marketed?	Yes	509	43.4
	No	663	56.6
Do you know about the National Pharmacovigilance and Drug Safety Center administered by the SFDA?	Yes	1,016	86.7
	No	156	13.3
Are you familiar with the adverse drug reaction reporting form for healthcare professionals (Form No. ADR-1)?	Yes	830	70.8
	No	342	29.2
Do you know where to get the adverse drug reaction reporting form (Form No. ADR-1) from?	Yes	744	63.5
	No	428	36.5
Do you know to whom you should submit the drug reaction reports?	Yes	951	81.1
	No	221	18.9
How do you rate your knowledge and training about the method of reporting adverse drug reactions to the SFDA?	1	23	2.0
	2	46	3.9
	3	262	22.4
	4	463	39.5
	5	378	32.3
What do you think is the purpose of the National Pharmacovigilance and Drug Safety Center that is administered by the SFDA?			
To enhance patients’ safety concerning the use of drugs	Yes	1,133	96.7
	No	39	3.3
To early detect and prevent frequent adverse drug reactions	Yes	1,130	96.4
	No	42	3.6
To identify predisposing factors to adverse drug reactions	Yes	1,092	93.2
	No	80	6.8
To identify rare adverse drug reactions	Yes	1,097	93.6
	No	75	6.4
To estimate the prevalence and incidence of adverse drug reactions	Yes	1,113	95.0
	No	59	5.0
To communicate with the international institutions working in pharmacovigilance	Yes	1,102	94.0
	No	70	6.0
To improve drug prescribing systems and regulations	Yes	1,096	93.5
	No	76	6.5
To access drug quality surveillance	Yes	1,091	93.1
	No	81	6.9
Which of the following scenarios would always be considered a severe adverse event by the SFDA?			
An adverse event that results in death	Yes	1,044	89.1
	No	128	10.9
An adverse event that results in hospitalization	Yes	1,046	89.2
	No	126	10.8
An adverse event that requires intervention to prevent permanent impairment/damage	Yes	1,037	88.5
	No	135	11.5
An adverse event that requires an emergency room visit	Yes	1,046	89.2
	No	126	10.8
An adverse event that results in congenital anomaly/birth defect	Yes	1,053	89.8
	No	119	10.2
An adverse event that is a life-threatening condition by a healthcare professional	Yes	1,064	90.8
	No	108	9.2
What aspect of adverse drug reaction deserves reporting by community pharmacists?			
The seriousness of the adverse drug reaction	Yes	1,028	87.7
	No	144	12.2
The unusualness of the adverse drug reaction	Yes	899	76.7
	No	273	23.2
Adverse drug reaction for the new drug only	Yes	735	62.7
	No	437	37.2
All types of adverse drug reactions	Yes	1,001	85.4
	No	171	14.5

The respondents rated all the eight knowledge-related statements about the purpose of the National Pharmacovigilance and Drug Safety Center as high on a 2-point Likert scale. The majority considered the scenario of “an adverse event that is a life-threatening condition by a healthcare professional” as a severe adverse event by the SFDA (1,064, 90.8%), followed by “an adverse event that results in congenital anomaly/birth defect” (1,053, 89.8%). The aspect of adverse event reporting that was rated the highest was the “seriousness of the adverse events” (1,028, 87.7%) (Table 2).

There was a statistically significant effect of age on the responses of pharmacists to the question “Do you know about the National Pharmacovigilance and Drug Safety Center administered by the SFDA?” (*p* < 0.05). In addition, there were significant sex-dependent differences in the community pharmacists’ perceived knowledge about the method of reporting ADRs to the SFDA (*p* < 0.05), and their responses to the questions “Do you think all serious drug reactions are known before a drug is marketed?” (*p* < 0.05) and “Do you know to whom you should submit the drug reaction reports?” (*p* < 0.05). Moreover, the perceived knowledge about the terms “pharmacovigilance” and “adverse drug reaction” was significantly affected by the educational level (*p* < 0.05), years of experience (*p* < 0.05), and professional classification (*p* < 0.05). Furthermore, there were significant differences by the educational level (*p* < 0.05) and years of experience (*p* < 0.05) in the responses of community pharmacists to the question “How do you rate your knowledge and training about the method of reporting adverse drug reactions to the SFDA?” Questions about their perceived knowledge of the ADR reporting form and to whom it was to be submitted were significantly affected by the years of experience (*p* < 0.01) and professional classification (*p* < 0.05). Work region and professional classification significantly influenced the response of the community pharmacists to the question on whether all serious adverse drug reactions were known before a drug was marketed (*p* < 0.05) (Supplementary Table S1).

### Attitude of Community Pharmacists Toward Pharmacovigilance and Adverse Drug Reaction Reporting

The attitude of the participants toward pharmacovigilance and ADR reporting is shown in Table 3. The majority agreed that ADR reporting was significant for patient care (1,103, 94.1%) and positively contributed to the national health (1,108, 94.5%). However, more than half of the participants considered the ADR reporting system to be too complex and time consuming to complete (665, 56.7%). The majority asked their customers/patients about ADRs (1,081, 92.2%), agreed that the pharmacists have a professional obligation to report ADRs (1,057, 90.2%), and agreed that the SFDA should implement more training programs for the healthcare professionals related to ADRs detection and reporting (1,112, 94.9%). Less than half agreed that reporting ADRs should be voluntary for community pharmacists (527, 45.0%). The majority also believed that they would be encouraged to report more ADRs if incentives were present (932, 79.5%).

**TABLE 3:** Attitudes of respondent pharmacists toward pharmacovigilance and ADR reporting.

Variable		Frequency (*n* = 1,172)	Percentage (%)
Do you think adverse drug reaction reporting is significant for patient care?	Agree	1,103	94.1
	Disagree	69	5.9
Do you think adverse drug reaction reporting has a positive contribution to our national health?	Agree	1,108	94.5
	Disagree	64	5.5
Do you think that the adverse drug reaction reporting system is too complex to fill out and time consuming?	Agree	665	56.7
	Disagree	507	43.3
Do you think the pharmacists should ask patients/customers about their adverse drug reactions?	Agree	1,081	92.2
	Disagree	91	7.8
Do you think that pharmacists have a professional obligation to report adverse drug reactions?	Agree	1,057	90.2
	Disagree	115	9.8
Do you think the SFDA should implement more training programs for healthcare professionals on adverse drug reaction detection and reporting?	Agree	1,112	94.9
	Disagree	60	5.1
In your opinion, to what extent the reporting of adverse drug reactions should be made mandatory for community pharmacists?	Mandatory	424	36.2
	Voluntary	527	45.0
	Not necessary to report	49	4.2
	Not sure	172	14.7
Would you be encouraged to report more adverse drug reactions if there were incentives?	Yes	932	79.5
	No	240	20.5

There were significant differences by age and educational level of the community pharmacists in their response to the question “In your opinion, to what extent the reporting of adverse drug reactions should be made mandatory for community pharmacists?” (*p* < 0.05). In addition, there were significant differences in the responses of the community pharmacists to the questions “Would you be encouraged to report more adverse drug reactions if there were incentives?” by age (*p* < 0.05), “Do you think that the adverse drug reaction reporting system is too complex to fill out and time consuming?” by sex (*p* < 0.05), and “Do you think adverse drug reaction reporting is significant for patient care?” by the educational level (*p* < 0.05) (Table 4).

**TABLE 4:** Attitudes of community pharmacists toward pharmacovigilance and adverse drug reaction reporting depending on the characteristics of the respondents.

Items	Comparisons				*p*-value
In your opinion, to what extent the reporting of adverse drug reactions should be made mandatory for community pharmacists?	Age				
	24–35 years	36–45 years	46–55 years	>55 years	<0.05
Mandatory	353 (36.4%)	62 (35.0%)	9 (42.9%)	0 (0.0%)	
Voluntary	433 (44.6%)	85 (48.0%)	9 (42.9%)	0 (0.0%)	
Not necessary to report	44 (4.5%)	5 (2.8%)	0 (0.0%)	0 (0.0%)	
Not sure	140 (14.4%)	25 (14.1%)	3 (14.3%)	4 (100.0%)	
Total	970 (100.0%)	177 (100.0%)	21 (100.0%)	4 (100.0%)	
Would you be encouraged to report more adverse drug reactions if there were incentives?	24–35 years	36–45 years	46–55 years	>55 years	
Yes	788 (81.2%)	123 (69.5%)	18 (85.7%)	3 (75.0%)	<0.05
No	182 (18.8%)	54 (30.5%)	3 (14.3%)	1 (25.0%)	
Total	970 (100.0%)	177 (100.0%)	21 (100.0%)	4 (100.0%)	
Do you think that the adverse drug reaction reporting system is too complex to fill out and time consuming?	Gender				
	Male		Female		
Agree	646 (57.4%)		19 (41.3%)		<0.05
Disagree	480 (42.6%)		27 (58.7%)		
Total	1,126 (100.0%)		46 (100.0%)		
Do you think adverse drug reaction reporting is significant for patient care?	Educational Level				
	B. Pharm	Pharm. D	Master	PhD	
Agree	905 (94.7%)	104 (89.7%)	15 (83.3%)	79 (96.3%)	<0.05
Disagree	51 (5.3%)	12 (10.3%)	3 (16.7%)	3 (3.7%)	
Total	956 (100.0%)	116 (100.0%)	18 (100.0%)	82 (100.0%)	
In your opinion, to what extent the reporting of adverse drug reactions should be made mandatory for the community pharmacists?	B. Pharm	Pharm. D	Master	PhD	
Mandatory	343 (35.9%)	45 (38.8%)	3 (16.7%)	33 (40.2%)	<0.05
Voluntary	434 (45.4%)	47 (40.5%)	13 (72.2%)	33 (40.2%)	
Not necessary to report	31 (3.2%)	14 (12.1%)	0 (0.0%)	4 (4.9%)	
Not sure	148 (15.5%)	10 (8.6%)	2 (11.1%)	12 (14.6%)	
Total	956 (100.0%)	116 (100.0%)	18 (100.0%)	82 (100.0%)	

### Practices of Community Pharmacists

The practices of the community pharmacists regarding pharmacovigilance and ADRs reporting are shown in Table 5. Most respondents had served more than 100 customers/patients daily (699, 59.6%). Among the respondents, 33.9% had never reported an ADR to the SFDA; however, 25.9% had reported an ADR more than three times. The frequency of observing ADRs in their customers/patients was rated as “sometimes” (549, 46.8%) and “always” (110, 9.4%). The respondents were asked to rate the frequency of ADR reporting for 18 different products on a 2-point Likert scale. ADRs were most frequently associated with analgesic agents (790, 67.4%), followed by gastrointestinal agents (591, 50.4%), cardiovascular agents (575, 49.1%), and medical devices and supplies (558, 47.6%). ADRs were most commonly noted in people with chronic diseases (1,079, 92.1%), followed by older adults (1,034, 88.2%) and pregnant women (952, 81.2%), whereas they were least frequent in adult men (693, 59.1%) (Table 5).

**TABLE 5:** Pharmacovigilance and ADR reporting practices of respondent pharmacists.

Variable		Frequency (*n* = 1,172)	Percentage (%)
How many patients/customers per pharmacist are served daily?	<20	21	1.8
	20–50	71	6.1
	51–100	381	32.5
	>100	699	59.6
How often do you see adverse drug reactions among patients/customers?	Always (100%)	110	9.4
	Often (>50%)	152	13.0
	Sometimes (<50%)	549	46.8
	Rarely (≈20%)	326	27.8
	Never (0%)	35	3.0
Have you ever reported an adverse drug reaction to the SFDA?	Yes (>3 times)	304	25.9
	Yes (2 or more times)	248	21.2
	Yes (1 time)	223	19.0
	No (0 time)	397	33.9
Have you ever reported an adverse drug reaction related to the following?			
Herbal products and supplements	Yes	518	44.1
	No	654	55.8
Cosmetic products	Yes	493	42.0
	No	679	57.9
Vaccines	Yes	341	29.0
	No	831	70.9
Cardiovascular agents	Yes	575	49.0
	No	597	50.9
Respiratory tract agents	Yes	532	45.3
	No	640	54.6
Gastrointestinal agents	Yes	591	50.4
	No	581	49.5
Neurological agents	Yes	516	44.0
	No	656	55.9
Psychiatric agents	Yes	522	44.5
	No	650	55.4
Hormonal agents	Yes	439	37.4
	No	733	62.5
Diabetic agents	Yes	528	45.0
	No	644	54.9
Genitourinary agents	Yes	433	36.9
	No	739	63.0
Immunological agents	Yes	389	33.1
	No	783	66.8
Bone and joints agents	Yes	475	40.5
	No	697	59.4
Anti-infective agents	Yes	515	43.9
	No	657	56.0
Eyes, ears, nose, and throat agents	Yes	450	38.3
	No	722	61.6
Medical devices and supplies	Yes	558	47.6
	No	614	52.3
Analgesic agents	Yes	790	67.4
	No	382	32.5
Dermatological agents	Yes	501	42.7
	No	671	57.2
From your experience, who is more likely to have adverse drug reactions?			
Babies (<6 years)	Yes	839	71.5
	No	333	28.4
Children (6–18 years)	Yes	774	66.0
	No	398	33.9
Adults (men)	Yes	693	59.1
	No	479	40.8
Adults (women)	Yes	716	61.0
	No	456	38.9
Pregnant women	Yes	952	81.2
	No	220	18.7
Old people	Yes	1,034	88.2
	No	138	11.7
People with chronic disease	Yes	1,079	92.0
	No	93	7.9

There were statistically significant effects of age, sex, years of experience, and professional classifications of the community pharmacists on their response to the question “How often do you see adverse drug reactions among patients?” (*p* < 0.05). In addition, sex, educational level, years of experience, and professional classifications influenced the responses of the community pharmacists to the question “Have you ever reported an adverse drug reaction to the SFDA?” (*p* < 0.05) (Table 6).

**TABLE 6:** Pharmacovigilance and adverse drug reaction reporting practices of community pharmacists depending on the characteristics of the respondents.

Items	Comparisons				*p*-value
How often do you see adverse drug reactions among patients?	Age				
	24–35 years	36–45 years	46–55 years	>55 years	
Always	99 (10.2%)	7 (4.0%)	4 (19.0%)	0 (0.0%)	<0.05
Often	116 (12.0%)	33 (18.6%)	3 (14.3%)	0 (0.0%)	
Sometimes	459 (47.3%)	76 (42.9%)	12 (57.1%)	2 (50.0%)	
Rarely	268 (27.6%)	56 (31.6%)	1 (4.8%)	1 (25.0%)	
Never	28 (2.9%)	5 (2.8%)	1 (4.8%)	1 (25.0%)	
Total	970 (100.0%)	177 (100.0%)	21 (100.0%)	4 (100.0%)	
Have you ever reported an adverse drug reaction to the SFDA?	Gender				
	Male		Female		
Yes (>3 times)	304 (27.0%)		0 (0.0%)		<0.05
Yes (2 or more times)	245 (21.8%)		3 (6.5%)		
Yes (1 time)	217 (19.3%)		6 (13.0%)		
No (0 time)	360 (32.0%)		37 (80.4%)		
Total	1,126 (100.0%)		46 (100.0%)		
How often do you see adverse drug reactions among patients?	Male		Female		
Always	107 (9.5%)		3 (6.5%)		<0.05
Often	149 (13.2%)		3 (6.5%)		
Sometimes	531 (47.2%)		18 (39.1%)		
Rarely	308 (27.4%)		18 (39.1%)		
Never	31 (2.8%)		4 (8.7%)		
Total	1,126 (100.0%)		46 (100.0%)		
Have you ever reported an adverse drug reaction to the SFDA?	Educational level				
	B. Pharm	Pharm. D	Master	PhD	
Yes (>3 times)	262 (27.4%)	17 (14.7%)	4 (22.2%)	21 (25.6%)	<0.05
Yes (2 or more times)	199 (20.8%)	21 (18.1%)	7 (38.9%)	21 (25.6%)	
Yes (1 time)	181 (18.9%)	19 (16.4%)	2 (11.1%)	21 (25.6%)	
No (0 time)	314 (32.8%)	59 (50.9%)	5 (27.8%)	19 (23.2%)	
Total	956 (100.0%)	116 (100.0%)	18 (100.0%)	82 (100.0%)	
Have you ever reported an adverse drug reaction to the SFDA?	Years of experience				
	<5 years	5–10 years	11–20 years	>20 years	
Yes (>3 times)	34 (10.9%)	180 (29.5%)	85 (35.7%)	5 (45.5%)	<0.05
Yes (2 or more times)	48 (15.4%)	145 (23.7%)	52 (21.8%)	3 (27.3%)	
Yes (1 time)	63 (20.2%)	122 (20.0%)	36 (15.1%)	2 (18.2%)	
No (0 time)	167 (53.5%)	164 (26.8%)	65 (27.3%)	1 (9.1%)	
Total	312 (100.0%)	611 (100.0%)	238 (100.0%)	11 (100.0%)	
How often do you see adverse drug reactions among patients?	<5 years	5–10 years	11–20 years	>20 years	<0.05
Always	31 (9.9%)	60 (9.8%)	16 (6.7%)	3 (27.3%)	
Often	36 (11.5%)	76 (12.4%)	39 (16.4%)	1 (9.1%)	
Sometimes	126 (40.4%)	306 (50.1%)	110 (46.2%)	7 (63.6%)	
Rarely	101 (32.4%)	157 (25.7%)	68 (28.6%)	0 (0.0%)	
Never	18 (5.8%)	12 (2.0%)	5 (2.1%)	0 (0.0%)	
Total	312 (100.0%)	611 (100.0%)	238 (100.0%)	11 (100.0%)	
Have you ever reported an adverse drug reaction to the SFDA?	Professional classification				
	Pharmacist	Senior pharmacist	Consultant pharmacist		
Yes (>3 times)	145 (21.8%)	135 (30.4%)	24 (38.1%)		<0.05
Yes (2 or more times)	107 (16.1%)	123 (27.7%)	18 (28.6%)		
Yes (1 time)	123 (18.5%)	93 (20.9%)	7 (11.1%)		
No (0 time)	290 (43.6%)	93 (20.9%)	14 (22.2%)		
Total	665 (100.0%)	444 (100.0%)	63 (100.0%)		
How often do you see adverse drug reactions among patients?	Pharmacist	Senior pharmacist	Consultant pharmacist		
Always	56 (8.4%)	45 (10.1%)	9 (14.3%)		<0.05
Often	90 (13.5%)	50 (11.3%)	12 (19.0%)		
Sometimes	297 (44.7%)	222 (50.0%)	30 (47.6%)		
Rarely	193 (29.0%)	122 (27.5%)	11 (17.5%)		
Never	29 (4.4%)	5 (1.1%)	1 (1.6%)		
Total	665 (100.0%)	444 (100.0%)	63 (100.0%)		

## Discussion

The present study evaluated the knowledge, attitudes, and practices of community pharmacists regarding pharmacovigilance and ADR reporting in Saudi Arabia. To the best of our knowledge, this study is the first to assess current practices in community pharmacies regarding pharmacovigilance and ADR reporting in different regions of Saudi Arabia. The results of our survey indicated that the majority of community pharmacists were aware of pharmacovigilance and ADR reporting. Their attitudes to ADR reporting were favorable. It is believed that ADR reporting must be made compulsory for all community pharmacists.

In contrast to studies conducted in Al Riyadh and Ash Sharqiyah, our study shows that community pharmacists have high awareness and knowledge regarding pharmacovigilance and methods of reporting ADRs (Bawazir, 2006; Khan, 2013; Mahmoud et al., 2014; AlRuthia et al., 2018; Al Doughan et al., 2019). Most of the respondents had 5–10 years of working experience with a full-time contract and had served more than 100 patients/customers daily. The satisfactory level of awareness might be explained by their years of experience as well as the efforts of the SFDA to establish a national platform, the National Pharmacovigilance Center (Hadi et al., 2013). This national platform aims to enforce regulations to improve current practices and adopt good pharmacy practice standards and guidelines for pharmacovigilance, as well as ADR detection and reporting processes in community pharmacists (Alshammari et al., 2017; Saudi Food and Drug Authority, 2021a).

The National Pharmacovigilance Center’s collective and continued efforts to initiate and encourage online and paper reporting of ADRs have been successful and have brought several benefits (Saudi Food and Drug Authority, 2021c). In a similar context, most participants in the present study have agreed that community pharmacists have a professional obligation to report ADRs. They were familiar with the ADR reporting form (Form No. ADR-1), knew where to obtain the form, and to whom they should submit it. However, they also agreed that the SFDA should implement more continuous training programs for healthcare professionals regarding ADR detection and reporting system.

The majority agreed that ADR reporting is important for patient safety and contributes positively to national health. These results are consistent with those of previous studies (Bawazir, 2006; Khan, 2013; Mahmoud et al., 2014; AI Doughan et al., 2019). The majority (90%) of the respondents agreed that pharmacists should ask their patients/customers about ADRs and 36% indicated that reporting ADRs should be made compulsory for community pharmacists.

The good level of knowledge and positive attitudes reported in the current study are comparable with responses of community pharmacists in other countries, namely, the United Kingdom, Poland, Lebanon, and Yemen (Zimmermann et al., 2016; Al-Worafi et al., 2017; Hajj et al., 2018; Hughes and Weiss, 2019). In addition, pharmacists had the highest level of knowledge and most positive attitudes toward pharmacovigilance and ADR reporting among all healthcare professionals in many countries. In Ireland, pharmacists had higher knowledge and awareness of ADR reporting than other healthcare practitioners (O’Callaghan et al., 2018). In addition, pharmacists and pharmacist technicians exhibited the highest rate of pharmacovigilance awareness among healthcare providers in Saudi Arabia (Almandil, 2016). The level of knowledge and attitudes of the respondents in our study were better than those reported for nursing and dentistry students (Sivadasan et al., 2014; Khan et al., 2015). In particular, Sivadasan et al. (2014) stated that the level of knowledge, understanding, and awareness of pharmacovigilance and ADR reporting was better in pharmacy students than in medical students. A study by Khan et al. (2015) found that pharmacy students had better knowledge and more positive attitudes toward handling and reporting ADRs than medical students did.

Most of the participants in the current study reported that receiving an incentive would encourage them to report more ADRs. Some studies have also reported the favorable impact of various incentives on ADR reporting (Pedrós et al., 2009; Gonzalez-Gonzalez et al., 2013; Chang et al., 2017; Ali et al., 2018). It should be noted that the SFDA periodically publicly acknowledges and commends community pharmacies for their commitment to drug safety standards by monitoring ADRs and reporting (Saudi Food and Drug Authority, 2021b). Although the respondents in this study expressed good knowledge and positive attitudes toward pharmacovigilance and ADR reporting, their practice of ADR reporting was unsatisfactory and did not reflect their knowledge and attitude. This may be due to different factors, such as the high number of patients served by each pharmacist, complexity of the ADR reporting system, time factor, lack of training programs regarding ADR detection and reporting provided by the SFDA to healthcare professionals, and the absence of incentives provided to the pharmacist as encouragement to enhance ADR reporting. Underreporting of ADRs by pharmacists is common not only in Saudi Arabia but also globally. The reasons for underreporting vary in different countries, for example, the lack of time was considered the most significant reporting barrier in Australia (Li et al., 2018), whereas in Germany, the lack of good training and long forms to complete were considered as dominant negative factors (Laven et al., 2018). There is a critical need globally to resolve the reporting barriers to improve ADR reporting.

The respondents in the present study disclosed that the frequency of observing ADRs in patients/customers can be categorized as “sometimes,” i.e., in less than half of cases. ADRs were most frequently observed with analgesic agents, in elderly patients/customers, and in people with chronic diseases. These results agree with those of previous studies (Almubark et al., 2020). Although this study assessed the national knowledge, practices, and attitudes of community pharmacists toward pharmacovigilance and ADR reporting in a sample of pharmacists of a sufficient size, it was an Internet-based survey that might have been affected by reporting bias. Additionally, the respondents may not have been willing to reveal deficiencies in their practices.

The sample in the present study disclosed that the frequency of observing ADRs in their patients/customers as “sometimes”. The highest category was analgesic agents, elderly patients/customers, and people with a chronic disease. These results are in agreement with prior research (Almubark et al., 2020). Although the study reported the national knowledge, practice, and attitude of community pharmacists toward pharmacovigilance and ADRs reporting with a sufficient sample size, it was an Internet-based survey which might be affected by some reporting bias. In addition, they may not have been willing to reveal their practice deficiencies.

### Practical Implications

The results of this study have several practical implications. More training in pharmacovigilance and ADR reporting is required, given the importance of improving the understanding of and need to minimize drug-related problems. Lecture-based seminars on pharmacovigilance and ADR reporting may enhance the knowledge, attitudes, and practices of healthcare students. The familiarity of students with ADRs and methods to assess their cause and severity needs to be increased. The essential elements of the comprehensive pharmacovigilance curriculum were developed by the WHO and International Society of Pharmacovigilance to assist integration in the healthcare school curriculum. The integration of these initiatives is likely to improve the level of knowledge of community pharmacists regarding pharmacovigilance.

## Conclusion

Most community pharmacists in Saudi Arabia are knowledgeable and have good attitudes and practices regarding pharmacovigilance and ADR reporting. Our findings illustrate an improvement in the knowledge, attitudes, and practices of the community pharmacists regarding pharmacovigilance and ADR reporting. The SFDA should implement good pharmacy practice guidelines and standards and adopt continuous educational programs to enhance the current practices of community pharmacists regarding pharmacovigilance and ADR reporting.

## Data Availability

The original contributions presented in the study are included in the article/Supplementary Material, further inquiries can be directed to the corresponding author.
