# Supplementary material for: A National Survey of Community Pharmacists’ Viewpoints About Pharmacovigilance and Adverse Drug Reaction Reporting in Saudi Arabia
Source: Front Pharmacol. 2022 May 26;13:819551. doi: 10.3389/fphar.2022.819551 (PMC9204278; doi:10.3389/fphar.2022.819551)
Supplement: Supplementary file 1 [file Table1.docx]

**Table S1**. Knowledge of Community Pharmacist About Pharmacovigilance and Adverse Drug Reaction Reporting Depending on the Characteristics of the Respondents

| **Items** | **Comparisons** | | | | | | ***P-value*** |
| --- | --- | --- | --- | --- | --- | --- | --- |
| **Do you know about the National Pharmacovigilance and Drug Safety Center administered by the SFDA?** | **Age** | | | | | |  |
|  | **24–35 years** | **36–45**  **years** | | **46–55**  **years** | | **>55 years** |  |
| Yes | 828  (85.4%) | 164  (92.7%) | | 21  (100.0%) | | 3  (75.0%) | <.05 |
| No | 142  (14.6%) | 13  (7.3%) | | 0  (0.0%) | | 1  (25.0%) |  |
| Total | 970  (100.0%) | 177  (100.0%) | | 21  (100.0%) | | 4  (100.0%) |  |
| **Do you think all serious adverse drug reactions are known before a drug is marketed?** | **Gender** | | | | | |  |
|  | **Male** | | | **Female** | | |  |
| Yes | 499 (44.3%) | | | 10 (21.7%) | | | <.05 |
| No | 627 (55.7%) | | | 36 (78.3%) | | |  |
| Total | 1,126 (100.0%) | | | 46 (100.0%) | | |  |
| **How do you rate your knowledge and training about the method of reporting adverse drug reactions to the SFDA?** | **Male** | | | **Female** | | |  |
| 1 | 17 (1.5%) | | | 6 (13.0%) | | | <.05 |
| 2 | 43 (3.8%) | | | 3 (6.5%) | | |  |
| 3 | 253 (22.5%) | | | 9 (19.6%) | | |  |
| 4 | 449 (39.9%) | | | 14 (30.4%) | | |  |
| 5 | 364 (32.3%) | | | 14 (30.4%) | | |  |
| Total | 1,126 (100.0%) | | | 46 (100.0%) | | |  |
| **Do you know to whom you should submit the drug reaction reports?** | **Male** | | | **Female** | | |  |
| Yes | 919 (81.6%) | | | 32 (69.6%) | | | <.05 |
| No | 207 (18.4%) | | | 14 (30.4%) | | |  |
| Total | 1,126 (100.0%) | | | 46 (100.0%) | | |  |
| **How familiar are you with the term "pharmacovigilance?** | **Educational Level** | | | | | |  |
|  | **B. Pharm** | **Pharm. D** | | **Master** | | **PhD** |  |
| Very familiar - I've a complete understanding | 500 (52.3%) | 50  (43.1%) | | 8  (44.4%) | | 38  (46.3%) | <.05 |
| Familiar - I've a basic understanding | 404 (42.3%) | 43  (37.1%) | | 9  (50.0%) | | 33  (40.2%) |  |
| Heard of the term - cannot define it | 34  (3.6%) | 13  (11.2%) | | 1  (5.6%) | | 8  (9.8%) |  |
| Never heard of the term | 18 (1.9%) | 10  (8.6%) | | 0  (0.0%) | | 3  (3.7%) |  |
| Total | 956 (100.0%) | 116  (100.0%) | | 18  (100.0%) | | 82  (100.0%) |  |
| **How familiar are you with the term "adverse drug reaction"?** | **B. Pharm** | **Pharm. D** | | **Master** | | **PhD** |  |
| Very familiar - I've a complete understanding | 546 (57.1%) | 64  (55.2%) | | 8  (44.4%) | | 31 (37.8%) | <.05 |
| Familiar - I've a basic understanding | 362 (37.9%) | 35  (30.2%) | | 10  (55.6%) | | 39 (47.6%) |  |
| Heard of the term - cannot define it | 32  (3.3%) | 11  (9.5%) | | 0  (0.0%) | | 10 (12.2%) |  |
| Never heard of the term | 16 (1.7%) | 6  (5.2%) | | 0  (0.0%) | | 2  (2.4%) |  |
| Total | 956 (100.0%) | 116  (100.0%) | | 18  (100.0%) | | 82 (100.0%) |  |
| **How do you rate your knowledge and training about the method of reporting adverse drug reactions to the SFDA?** | **B. Pharm** | **Pharm. D** | | **Master** | | **PhD** |  |
| 1 | 14 (1.5%) | 9  (7.8%) | | 0  (0.0%) | | 0  (0.0%) | <.05 |
| 2 | 40 (4.2%) | 6  (5.2%) | | 0  (0.0%) | | 0  (0.0%) |  |
| 3 | 222 (23.2%) | 21  (18.1%) | | 6  (33.3%) | | 13  (15.9%) |  |
| 4 | 378 (39.5%) | 43 (37.1%) | | 4 (22.2%) | | 38 (46.3%) |  |
| 5 | 302 (31.6%) | 37  (31.9%) | | 8  (44.4%) | | 31 (37.8%) |  |
| Total | 956 (100.0%) | 116 (100.0%) | | 18 (100.0%) | | 82 (100.0%) |  |
| **How familiar are you with the term "pharmacovigilance?** | **Years of Experience** | | | | | |  |
|  | **<5**  **years** | **5–10**  **years** | | **11–20**  **years** | | **>20 years** |  |
| Very familiar - I've a complete understanding | 119  (38.1%) | 332  (54.3%) | | 139  (58.4%) | | 6  (54.5%) | <.05 |
| Familiar - I've a basic understanding | 153 (49.0%) | 241  (39.4%) | | 91  (38.2%) | | 4  (36.4%) |  |
| Heard of the term - cannot define it | 28  (9.0%) | 23  (3.8%) | | 4  (1.7%) | | 1  (9.1%) |  |
| Never heard of the term | 12  (3.8%) | 15  (2.5%) | | 4  (1.7%) | | 0  (0.0%) |  |
| Total | 312  (100.0%) | 611  (100.0%) | | 238  (100.0%) | | 11  (100.0%) |  |
| **How familiar are you with the term "adverse drug reaction"?** | **<5**  **years** | **5–10**  **years** | | **11–20**  **years** | | **>20 years** |  |
| Very familiar - I've a complete understanding | 156  (50.0%) | 356  (58.3%) | | 131  (55.0%) | | 6  (54.5%) | <.05 |
| Familiar - I've a basic understanding | 123  (39.4%) | 223  (36.5%) | | 97  (40.8%) | | 3  (27.3%) |  |
| Heard of the term - cannot define it | 22  (7.1%) | 23  (3.8%) | | 8  (3.4%) | | 0  (0.0%) |  |
| Never heard of the term | 11  (3.5%) | 9  (1.5%) | | 2  (0.8%) | | 2  (18.2%) |  |
| Total | 312  (100.0%) | 611  (100.0%) | | 238  (100.0%) | | 11  (100.0%) |  |
| **Do you know about the National Pharmacovigilance and Drug Safety Center administered by the SFDA?** | **<5**  **years** | **5–10**  **years** | | **11–20**  **years** | | **>20 years** |  |
| Yes | 249  (20.2%) | 540  (88.4%) | | 216  (90.8%) | | 11  (100%) | <.05 |
| No | 63  (20.2%) | 71  (11.6%) | | 22  (9.2%) | | 0  (0.0%) |  |
| Total | 312  (100.0%) | 611  (100.0%) | | 238  (100.0%) | | 11  (100.0%) |  |
| **Do you know where to get the adverse drug reaction reporting form (Form NO. ADR-1) from?** | **<5**  **years** | **5–10**  **years** | | **11–20**  **years** | | **>20 years** |  |
| Yes | 175  (56.1%) | 397  (65.0%) | | 165  (69.3%) | | 7  (63.6%) | <.05 |
| No | 137  (43.9%) | 214  (35.0%) | | 73  (30.7%) | | 4  (36.4%) |  |
| Total | 312  (100.0%) | 611  (100.0%) | | 238  (100.0%) | | 11  (100.0%) |  |
| **Do you know to whom you should submit the drug reaction reports?** | **<5**  **years** | **5–10**  **years** | | **11–20**  **years** | | **>20 years** |  |
| Yes | 229  (73.4%) | 513  (84.0%) | | 199  (83.6%) | | 10  (90.9%) | <.05 |
| No | 83  (26.6%) | 98  (16.0%) | | 39  (16.4%) | | 1  (9.1%) |  |
| Total | 312  (100.0%) | 611  (100.0%) | | 238  (100.0%) | | 11  (100.0%) |  |
| **How familiar are you with the term "pharmacovigilance?** | **Professional Classification** | | | | | |  |
|  | **Pharmacist** | | **Senior pharmacist** | | **Consultant pharmacist** | |  |
| Very familiar - I've a complete understanding | 302  (45.4%) | | 251  (56.5%) | | 43  (68.3%) | | <.05 |
| Familiar - I've a basic understanding | 306  (46.0%) | | 166  (37.4%) | | 17  (27.0%) | |  |
| Heard of the term - cannot define it | 35  (5.3%) | | 18  (4.1%) | | 3  (4.8%) | |  |
| Never heard of the term | 22  (3.3%) | | 9  (2.0%) | | 0  (0.0%) | |  |
| Total | 665  (100.0%) | | 444  (100.0%) | | 63  (100.0%) | |  |
| **Do you think all serious adverse drug reactions are known before a drug is marketed?** | **Pharmacist** | | **Senior pharmacist** | | **Consultant pharmacist** | |  |
| Yes | 265  (39.8%) | | 213  (48.0%) | | 31  (49.2%) | | <.05 |
| No | 400  (60.2%) | | 231  (52.0%) | | 32  (50.8%) | |  |
| Total | 665  (100.0%) | | 444  (100.0%) | | 63  (100.0%) | |  |
| **Do you know about the National Pharmacovigilance and Drug Safety Center administered by the SFDA?** | **Pharmacist** | | **Senior pharmacist** | | **Consultant pharmacist** | |  |
| Yes | 558  (83.9%) | | 402  (90.5%) | | 56  (88.9%) | | <.05 |
| No | 107  (16.1%) | | 42  (9.5%) | | 7  (11.1%) | |  |
| Total | 665  (100.0%) | | 444  (100.0%) | | 63  (100.0%) | |  |
| **Are you familiar with the adverse drug reaction reporting form for health care professionals (Form NO. ADR-1)?** | **Pharmacist** | | **Senior pharmacist** | | **Consultant pharmacist** | |  |
| Yes | 448  (67.4%) | | 333  (75.0%) | | 49  (77.8%) | | <.05 |
| No | 217  (32.6%) | | 111  (25.0%) | | 14  (22.2%) | |  |
| Total | 665  (100.0%) | | 444  (100.0%) | | 63  (100.0%) | |  |
| **Do you know where to get the adverse drug reaction reporting form (Form NO. ADR-1) from?** | **Pharmacist** | | **Senior pharmacist** | | **Consultant pharmacist** | |  |
| Yes | 395  (59.4%) | | 303  (68.2%) | | 46  (73.0%) | | <.05 |
| No | 270  (40.6%) | | 141  (31.8%) | | 17  (27.0%) | |  |
| Total | 665  (100.0%) | | 444  (100.0%) | | 63  (100.0%) | |  |
| **Do you know to whom you should submit the drug reaction reports?** | **Pharmacist** | | **Senior pharmacist** | | **Consultant pharmacist** | |  |
| Yes | 509  (76.5%) | | 385  (86.7%) | | 57  (90.5%) | | <.05 |
| No | 156  (23.5%) | | 59  (13.3%) | | 6  (9.5%) | |  |
| Total | 665  (100.0%) | | 444  (100.0%) | | 63  (100.0%) | |  |
| **How familiar are you with the term "adverse drug reaction"?** | **Region** | | | | | |  |
|  | **Western Region** | **Central Region** | **Southern Region** | | **Eastern Region** | **Northern Region** |  |
| Very familiar - I've a complete understanding | 313  (55.3%) | 158  (56.4%) | 109  (50.9%) | | 51  (61.4%) | 18  (62.1%) | <.05 |
| Familiar - I've a basic understanding | 217  (38.3%) | 109  (38.9%) | 79  (36.9%) | | 31  (37.3%) | 10  (34.5%) |  |
| Heard of the term - cannot define it | 23  (4.1%) | 8  (2.9%) | 21  (9.8%) | | 0  (0.0%) | 1  (3.4%) |  |
| Never heard of the term | 13  (2.3%) | 5  (1.8) | 5  (2.3%) | | 1  (1.2%) | 0  (0.0%) |  |
| Total | 566  (100.0%) | 280  (100.0%) | 214  (100.0%) | | 83  (100.0%) | 29  (100.0%) |  |
| **Do you think all serious adverse drug reactions are known before a drug is marketed?** | **Western Region** | **Central Region** | **Southern Region** | | **Eastern Region** | **Northern Region** |  |
| Yes | 237  (41.9%) | 114  (40.7%) | 118  (55.1%) | | 31  (37.3%) | 9  (31.0%) | <.05 |
| No | 329  (58.1%) | 166  (59.3%) | 96  (44.9%) | | 52  (62.7%) | 20  (69.0%) |  |
| Total | 566  (100.0%) | 280  (100.0%) | 214  (100.0%) | | 83  (100.0%) | 29  (100.0%) |  |
